# BioTM buzz

**DOI:** 10.1002/btm2.10069

**Published:** 2017-07-17

**Authors:** Aaron C. Anselmo

**Affiliations:** ^1^ David H. Koch Institute for Integrative Cancer Research Massachusetts Institute of Technology, Cambridge, Massachusetts, 02139, USA

## Volume 2, Issue 2

### Reducing Adhesion Through Depletion and Addition

Improving the performance of nanoparticle drug delivery systems relies on understanding how nanoparticles interact with biological systems. Through this understanding, the development of nanoparticle drug delivery systems capable of navigating biological systems to improve targeted delivery can dramatically impact clinical care. In this issue, chemical engineers from the Omolola Eniola‐Adefeso lab (Univ. Michigan, Ann Arbor) define the role that individual immunoglobulin classes play in mediating adhesion of nanoparticles to endothelial cells under physiological flow conditions. Through systematic microfluidic studies involving the depletion and re‐addition of specific immunoglobulins, it was determined that nanoparticle adhesion to targeted endothelial cells was reduced due to the adsorption of IgA and IgM to nanoparticle surfaces. Importantly, these studies were performed using whole blood and plasma isolated from human donors, which highlight how immunoglobulin differences in a host's serum can potentially influence nanoparticle‐targeting efficacy in an individual. This study highlights how leveraging a systematic approach, focused on understanding how interactions between nanoparticles and biological systems can be used to better describe and design nanoparticle drug delivery systems.

DOI: 10.1002/btm2.10064

### Production and Performance: Teasing Apart Culture Parameters

Mesenchymal stem cell‐derived extracellular vesicles (MSC EVs) are a possible alternative to cell therapies due to their potentially superior pharmaceutical properties, safety profile, and regulatory pathway as compared to cells. As such, MSC EVs represent an exciting therapeutic modality for regenerative medicine provided that approaches to facilitate their reproducible and scalable biomanufacturing are developed. In this issue, researchers from the laboratories of Steven M. Jay and Kimberly M. Stroka (Fischell Dept. of Bioengineering, Univ. Maryland—College Park), describe how cell culture parameters influence MSC EV production and bioactivity. Specifically, the production of MSC EVs could be increased over 50‐fold per cell through culturing MSCs at low seeding densities. Increased frequency of collection was shown to yield further increases in EV production over a given time period. With regard to performance, passage number of EV‐producing MSCs was identified as crucial; EV vascularization bioactivity was retained up to passage 4 in culture before a significant decrease was observed at passage 5. Overall, this report outlines key culture parameters for EVs derived from MSCs as well as HEK293T cells, endothelial cells, and others. Identification of additional determinative parameters, and exploration of the molecular mechanisms that underlie these, will lay the foundation for development of rationally designed approaches to scalable and reproducible biomanufacturing of therapeutic MSC EVs toward their translation as a new class of biotherapeutics.

DOI: 10.1002/btm2.10065

## Recent literature

### Modulating Tumor Environments to Improve Nanoparticle Efficacy

A recently published research article from the Ralph Weissleder lab (MGH and Harvard Medical School describes an approach that utilizes radiation therapy to improve the efficacy of clinical nanoparticle formulations. This work builds on previous research that highlighted how tumor associated macrophages, which internalize intravenously injected nanoparticles, can act as active‐targeting drug reservoirs to deliver and distribute the internalized drug‐loaded nanoparticles to tumors. Here, the authors utilize computational modeling, intravital microscopy, and xenograft models in mice to describe the mechanism behind improving the efficacy of existing nanoparticle therapies by first modulating the tumor environment with radiation therapy. By treating tumors with radiation therapy, the tumor microenvironment is changed such that the infiltration of tumor‐associated macrophages can be increased by nearly 3.5 fold. The authors then show that macrophage‐internalized‐nanoparticles exhibit up to a 6‐fold increase in tumor accumulations following radiation therapy. This work describes the macrophage‐mediated mechanism behind using clinically relevant radiation therapy to modulate the tumor environment to improve the efficacy of clinical nanoparticle formulations. Given the widespread use of radiation therapy, the broader implications of this work point toward using this approach to improve existing nanoparticle therapies or toward the development of novel nanoparticle therapies designed to interact optimally with tumor associated macrophages for their targeted delivery to irradiated tumors.

*Miller et al., Sci Transl Med. 2017; doi:* 10.1126/scitranslmed.aal0225

